# Supplementary material for: 3′,8″-Dimerization Enhances the Antioxidant Capacity of Flavonoids: Evidence from Acacetin and Isoginkgetin
Source: Molecules. 2019 May 28;24(11):2039. doi: 10.3390/molecules24112039 (PMC6600363; doi:10.3390/molecules24112039)
Supplement: Supplementary file 1 [file molecules-24-02039-s001.zip › supplementary-proof/Suppl. 5 Figure S5.1-5.2 The certificate of analysis acacetin.pdf]

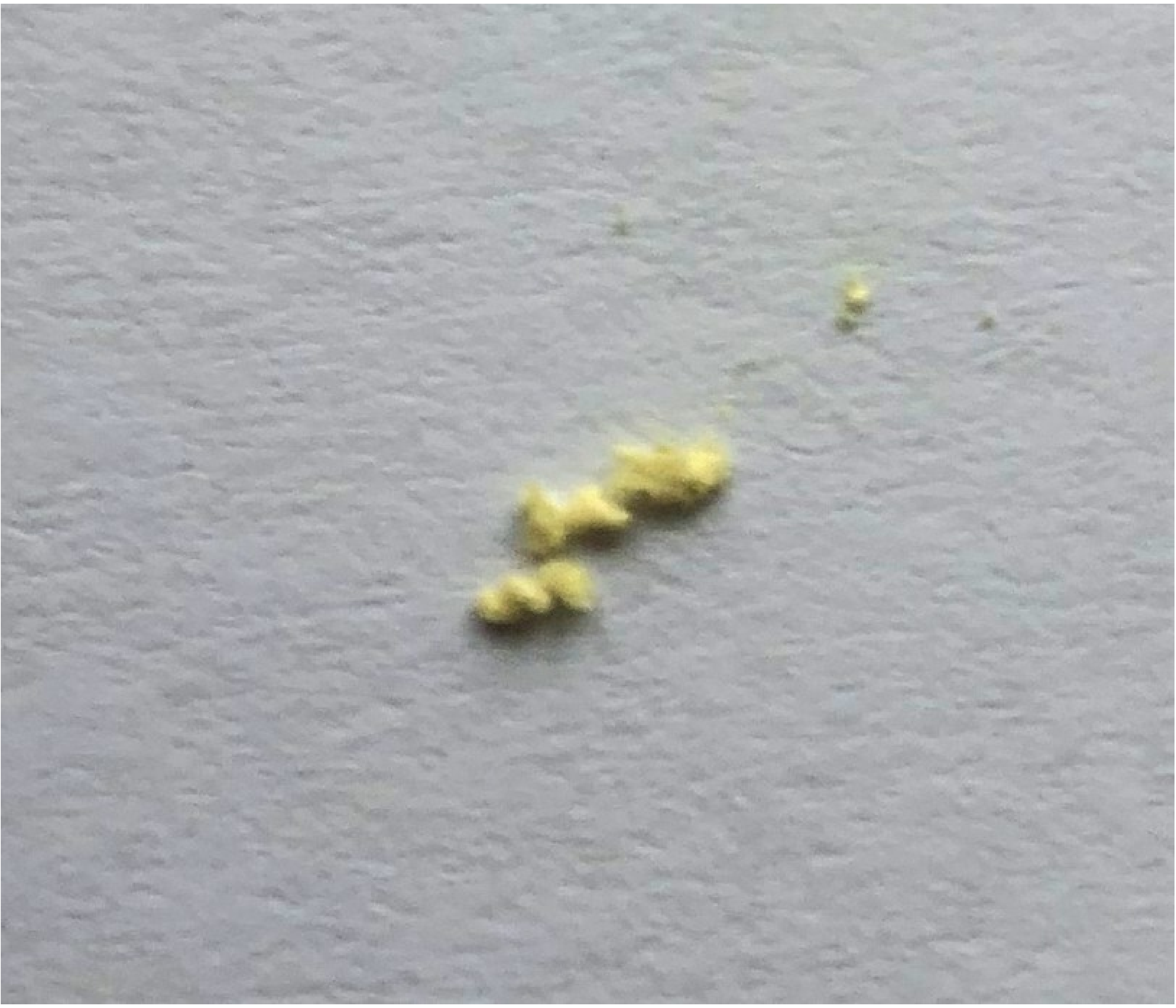

成都普瑞法科技开发有限公司
Chengdu Biopurify Phytochemicals Ltd.

文件编码（DOC NO.）：PRF-COA-BP011401

产品分析证书
Certificate of Analysis

中文名称：金合欢素

English Name：Acacetin
别名**(Alias):** Apigenin 4′-methyl ether; Linarigenin; Buddleoflavonol

产品编码**(Cat. No.):**BP0114
CAS Number: 480-44-4
分子式**(M. F.):** $C_{16}H_{12}O_5$
分子量**(M. W.)**: 284.267

批号**(Batch No.):** PRF8041842
报告日期**(Report date):** 2017-04-18

Structure

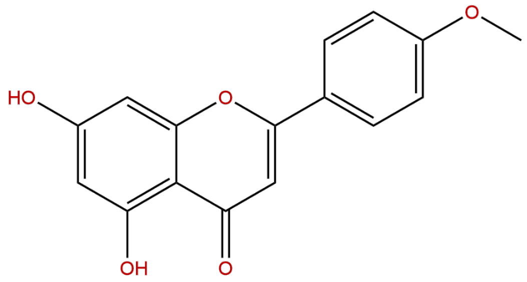

检验结果（**Analytical result**）：

检验项目（**Test Item**）	检验指标（**Specifications**）	检验结果（**Results**）
外观Appearance	Light pink powder	Light pink powder
干燥失重Loss on drying	＜3.0%	1.53%
纯度Purity (HPLC-DAD, 335nm)*	≥98.0%	98.67%
质谱Mass	284.2±1	Conforms
核磁NMR	Comply with the structure	Conforms

* 色谱图见附件（**Please find HPLC chromatography attached.**）

检测方法（**Test Method**）：**Column:** Thermo Hypersil GOLD AQ C18， 4.6*250mm， 5.0um; **Column temperature:** 30℃; **Detection Mode:** UV335nm； **Flow Rate:** 1.0ml/min; **Sample dissolution:** Methanol; **Mobile Phase: A**,0.1% Phosphoric acid in water **B**, Acetonitrile;**Gradient elution:** B, 35%-50%,15min.

贮存条件（**Storage**）:2~8℃, protected from light, keep package airproofed when not in use.

复测期（**Retest date**）:two years (2019-04-17) under conditions list above.

QC: Zhang Ling
Date: 2017-04-18

QA: Wu Qi
Date: 2017-04-18

备注**(Remarks)**: The sample solutions should be prepared and used on the same day, it is the best preparing the solutions immediately before use. If the solutons have to be made up in advance, it should be made as aliquots in tightly sealed vials at less than -20℃. Generally, these might be useable for up to two weeks.
In case of quality issue, please contact us within 15 days after receipt of the product.

Tel：+86-28-82633397 Fax：+86-28-82633165
http://www.phytopurify.com Email：sales@biopurify.com biopurify@gmail.com

数据文件: C:\QC\ACACETIN 20170418\ACACETIN PRF8041842.D
样品名称: PRF8041842

===

操作者 : DYZG ZHI BEI
仪器 : 仪器 1　　　　位置 : 样品瓶 1
进样日期 : 2017-4-18 13:02:08
　　　　进样量 : 10.0 µl
采集方法 : C:\QC\ACACETIN.M
最后修改 : 2017-4-18 12:53:58 : DYZG ZHI BEI
分析方法 : C:\QC\ACACETIN.M
最后修改 : 2017-4-18 13:52:14 : DYZG ZHI BEI
(调用后修改)
附加信息：峰已手动积分

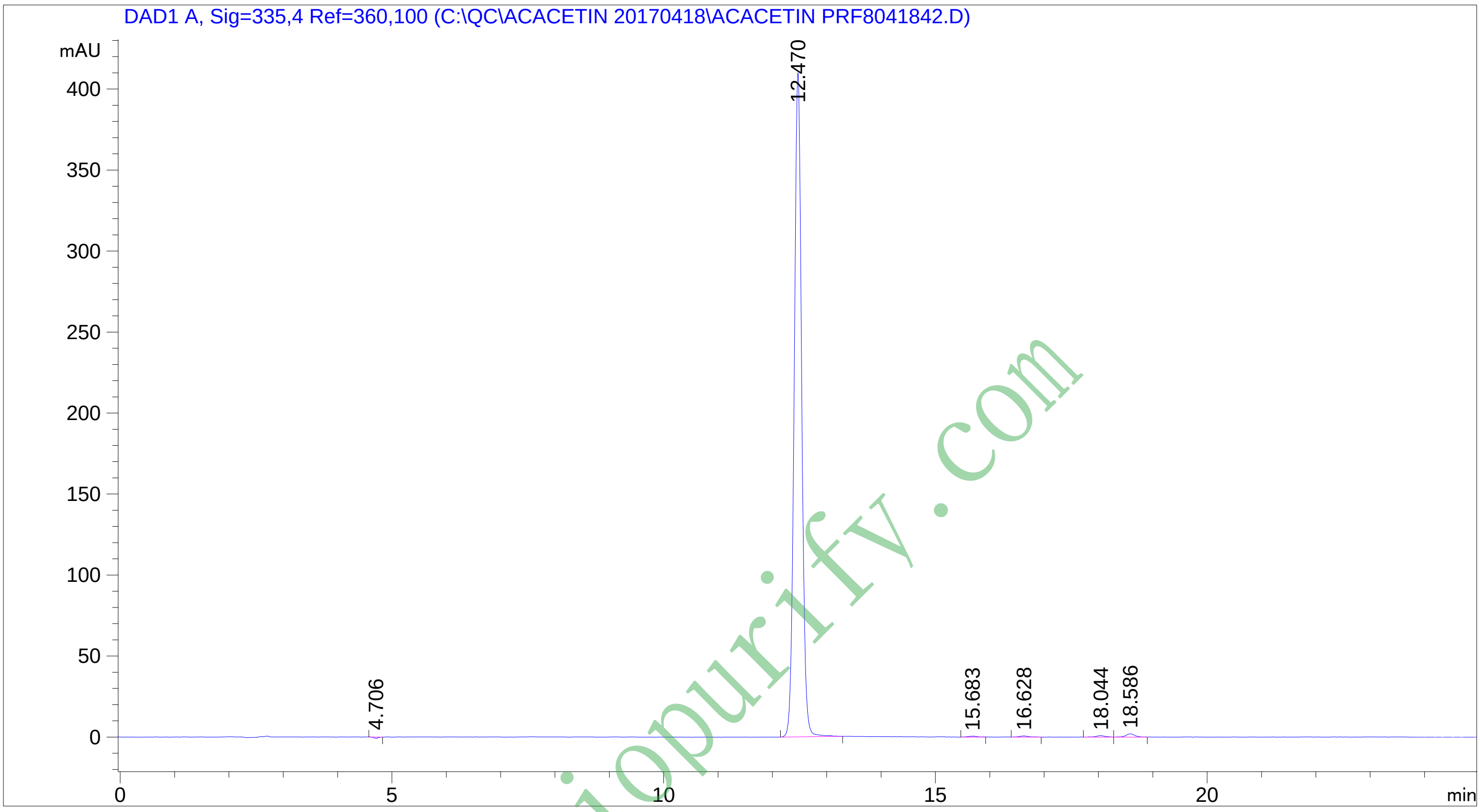

www.biopurify.com

===

面积百分比报告

===

排序 : 信号
乘积因子: : 1.0000
稀释因子: : 1.0000
内标使用乘积因子和稀释因子

信号 1: DAD1 A, Sig=335,4 Ref=360,100

峰 #	保留时间 [min]	类型	峰宽 [min]	峰面积 [mAU*s]	峰高 [mAU]	峰面积 %
1	4.706	MM R	0.0905	4.80431	8.12578e-1	0.1215
2	12.470	BV	0.1469	3901.06104	410.29437	98.6790
3	15.683	BV	0.1576	5.99660	5.39829e-1	0.1517
4	16.628	BB	0.1498	7.79535	7.11671e-1	0.1972
5	18.044	BV	0.1523	10.51488	8.87313e-1	0.2660
6	18.586	VV	0.1720	23.11108	2.01237	0.5846

总量 : 3953.28325 415.25813

===

*** 报告结束 ***
