# Supplementary material for: 3′,8″-Dimerization Enhances the Antioxidant Capacity of Flavonoids: Evidence from Acacetin and Isoginkgetin
Source: Molecules. 2019 May 28;24(11):2039. doi: 10.3390/molecules24112039 (PMC6600363; doi:10.3390/molecules24112039)
Supplement: Supplementary file 1 [file molecules-24-02039-s001.zip › supplementary-proof/Suppl. 6 Figure S6.1-2.2The certificate of analysis isoginkgetin.pdf]

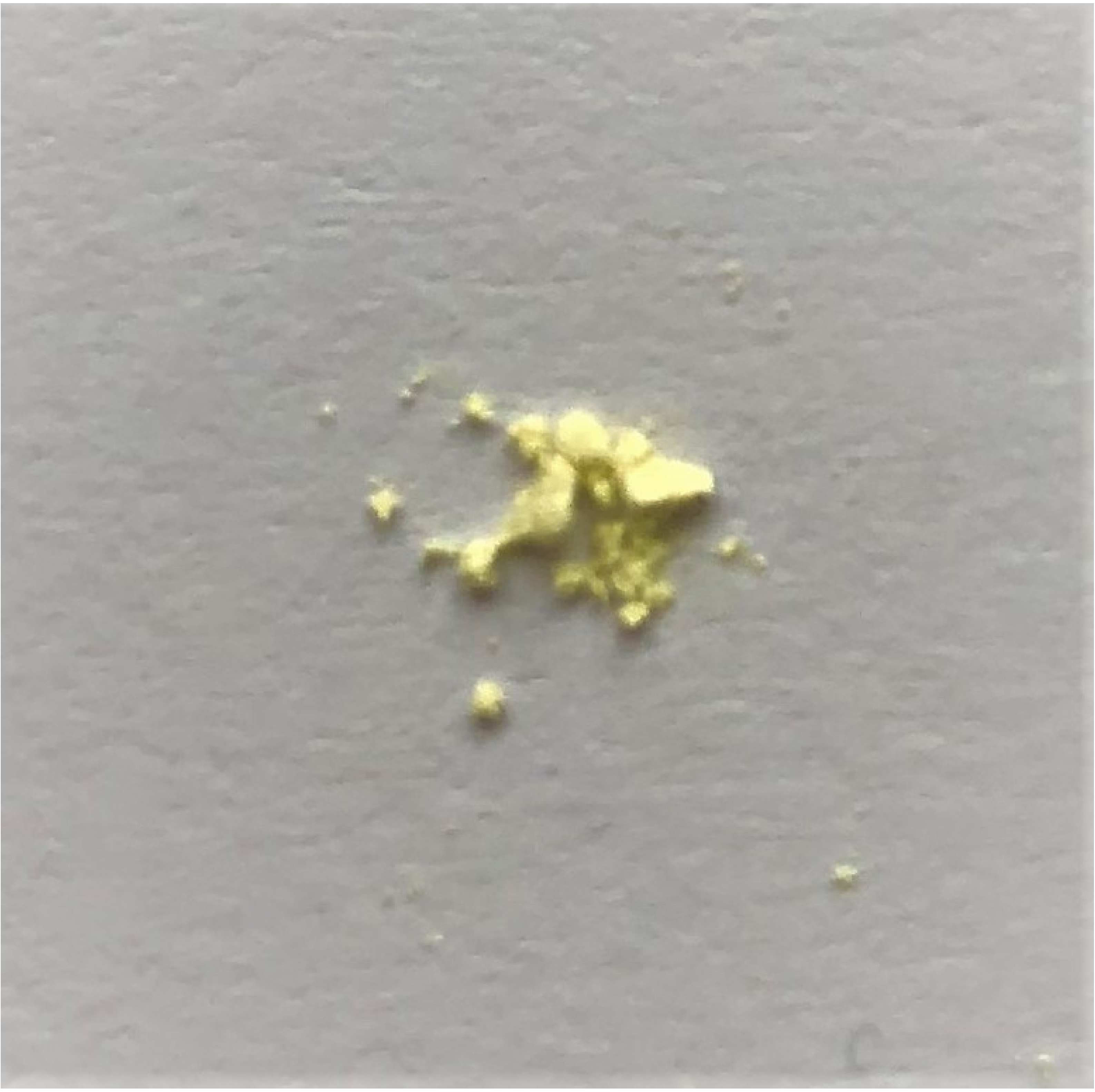

成都普瑞法科技开发有限公司
Chengdu Biopurify Phytochemicals Ltd.

文件编码（DOC NO.）：PRF-COA-BP078401

产品分析证书
Certificate of Analysis

中文名称：异银杏素

English Name：Isoginkgetin
别名(Alias):

产品编码(Cat. No.):BP0784
CAS Number: 548-19-6
分子式(M. F.): $C_{32}H_{22}O_{10}$
分子量(M. W.): 566.518

批号(Batch No.): PRF8060645
报告日期(Report date): 2017-06-06

Structure

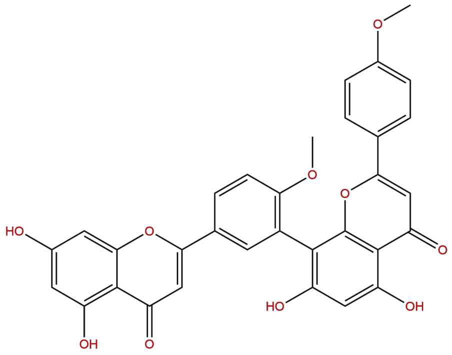

检验结果（Analytical result）：

**检验项目（Test Item）**	**检验指标（Specifications）**	**检验结果（Results）**
外观Appearance	Light yellow powder	Light yellow powder
干燥失重Loss on drying	＜3.0%	1.62%
纯度Purity (HPLC-DAD, 330nm)*	≥98.0%	98.80%
质谱Mass	566.5±1	Conforms
核磁NMR	Comply with the structure	Conforms

* 色谱图见附件（**Please find HPLC chromatography attached.**）

检测方法（**Test Method**）： **Column:** Thermo BDS Hypersil C18， 4.6*100mm， 2.4um; **Column temperature:** 30℃; **Detection Mode:** UV330nm;**Flow Rate:** 1.0ml/min;**Sample dissolution:** DMF dissolved and diluted with methanol; **Mobile Phase: A**, 0.1% phosphoric acid in water **B**, Acetonitrile;**Gradient elution: B**, 35%-45%,0min-15min;45%-45%,15min-35min.

贮存条件（**Storage**）:2~8℃, protected from light, keep package airproofed when not in use.

复测期（**Retest date**）:two years (2019-06-05) under conditions list above.

QC: Zhang Ling
Date: 2017-06-06

QA: Wu Qi
Date: 2017-06-06

备注(Remarks): The sample solutions should be prepared and used on the same day. It is the best preparing the solutions immediately before use. If the solutons have to be made up in advance, it should be made as aliquots in tightly sealed vials at less than -20℃. Generally, these might be useable for up to two weeks.
In case of quality issue, please contact us within 15 days after receipt of the product.

Tel：+86-28-82633397 Fax：+86-28-82633165
http://www.phytopurify.com Email：sales@biopurify.com biopurify@gmail.com

SAMPLE INFORMATION

Sample Name:	Isoginkgetin PRF8060645	Acquired By:	System
Sample Type:	Standard	Sample Set Name:	
Vial:	8	Acq. Method Set:	Isoginkgetin
Injection #:	1	Processing Method:	Samples
Injection Volume:	10.00 ul	Channel Name:	330.0nm
Run Time:	30.0 Minutes	Proc. Chnl. Descr.:	PDA 330.0 nm
Date Acquired:	2017-6-6 14:29:37 CST		
Date Processed:	2017-6-6 15:09:48 CST		

Auto-Scaled Chromatogram

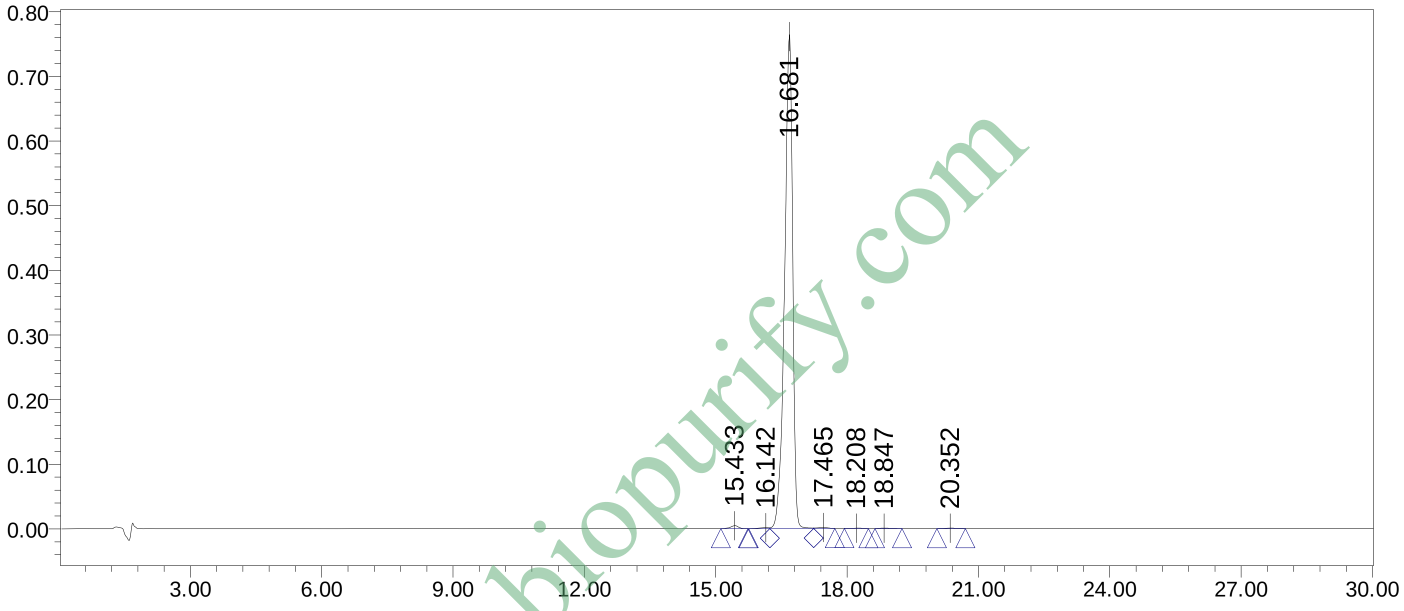

www.biopurify.com

Peak Results

	RT	Area	% Area	USP Plate Count	USP Resolution
1	15.433	53200	0.54	39599.70	
2	16.142	20696	0.21		
3	16.681	9718385	98.80	40269.50	
4	17.465	22853	0.23	31119.85	1.81
5	18.208	6101	0.06	42582.02	1.69
6	18.847	8062	0.08	48479.38	1.78
7	20.352	7264	0.07	42064.64	4.05
