# Supplementary material for: 3′,8″-Dimerization Enhances the Antioxidant Capacity of Flavonoids: Evidence from Acacetin and Isoginkgetin
Source: Molecules. 2019 May 28;24(11):2039. doi: 10.3390/molecules24112039 (PMC6600363; doi:10.3390/molecules24112039)
Supplement: Supplementary file 1 [file molecules-24-02039-s001.zip › supplementary-proof/supplementary3.pdf]

Suppl. 3 Dose response curves

3’,8”-Dimerization Enhances the Antioxidant Capacity of Flavonoids: Evidence from Acacetin and Isoginkgetin

Xican Li [1,2,*], Xiaojian Ouyang [1,2], Rongxin Cai [1,2], and Dongfeng Chen [3,4,*]

1 School of Chinese Herbal Medicine, Guangzhou University of Chinese Medicine, Waihuan East Road No. 232, Guangzhou Higher Education Mega Center, Guangzhou 510006, China; oyxiaojian55@163.com (X.O.); choi_roy@foxmail.com (R.C.)

2 Innovative Research & Development Laboratory of TCM, Guangzhou University of Chinese Medicine, Waihuan East Road No. 232, Guangzhou Higher Education Mega Center, Guangzhou 510006, China;

3 School of Basic Medical Science & Research Center of Basic Integrative Medicine, Guangzhou University of Chinese Medicine, Guangzhou 510006, China; chen888@gzucm.edu.cn (D.C.)

4 The Research Center of Basic Integrative Medicine, Guangzhou University of Chinese Medicine, Guangzhou 510006, China.

1. Superoxide anion (•O_2^-) scavenging assay

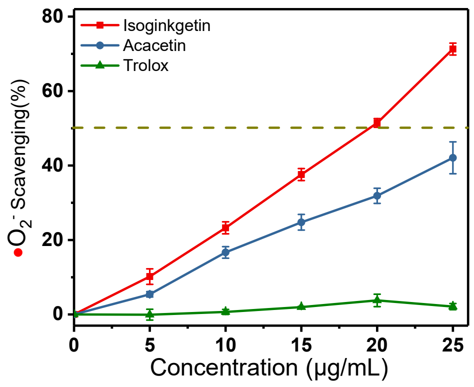

Figure S3.1: The dose response curves of acacetin and isoginkgetin in •O2⁻-inhibition assay. Each value is expressed as mean ± SD (n = 3).

Tab. S1 The comparison of IC_{50} values of acacetin, isoginkgetin and positive control in •O_2^- scavenging assay.

	Mean±SD μg/mL	Mean±SD μM
isoginkgetin	18.7±0.2	33.1±0.4 [a]
acacetin	30.5±3.2	107.1±11.3 [b]
Trolox	1243.6±39.5	4968.4±157.9

The IC_{50} value was defined as the lowest concentration with 50% radical inhibition or relative reducing power, calculated by linear regression analysis, and expressed as the mean ± SD (n = 3). The linear regression was analyzed by Origin 6.0 professional software. The IC_{50} values with different superscripts (a and b) between acacetin and isoginkgetin are significantly different ($p < 0.05$). Trolox was the positive control.

2. Cu^{2+}-reducing power assay

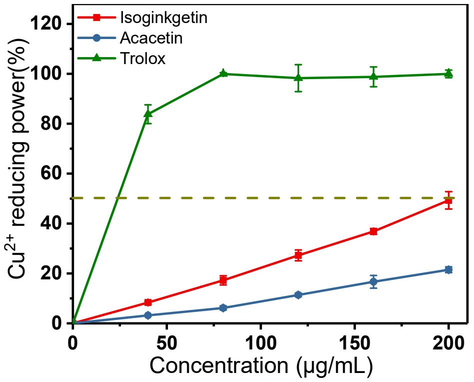

Figure S3.2: The dose response curves of acacetin and isoginkgetin in Cu^{2+}-reducing power assay. Each value is expressed as mean ± SD (n = 3).

Tab. S2 The comparison of IC_{50} values of acacetin, isoginkgetin and positive control in Cu^{2+}-reducing power assay.

	Mean±SD μg/mL	Mean±SD μM
isoginkgetin	207.9±12.1	367.1±21.4 [a]
acacetin	428.2±9.5	1506.2±33.4 [b]
Trolox	9.5±0.4	38.1±1.7

The IC_{50} value was defined as the lowest concentration with 50% radical inhibition or relative reducing power, calculated by linear regression analysis, and expressed as the mean ± SD (n = 3). The linear regression was analyzed by Origin 6.0 professional software. The IC_{50} values with different superscripts (a and b) between acacetin and isoginkgetin are significantly different ($p < 0.05$). Trolox was the positive control.

3. ABTS^{+}•-scavenging assay

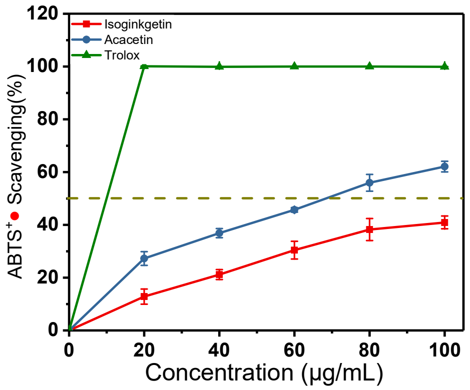

Figure S3.3: The dose response curves of acacetin and isoginkgetin in ABTS^{+}•-scavenging assay. Each value is expressed as mean ± SD (n = 3).

Tab. S3 The comparison of IC_{50} values of acacetin, isoginkgetin and positive control in ABTS^{+}•-scavenging assay.

	Mean±SD<br>μg/mL	Mean±SD<br>μM
isoginkgetin	118.6±8.6	209.3±15.1 [a]
acacetin	69.9±3.4	246.2±11.8 [b]
Trolox	5.7±0.1	22.6±0.3

The IC_{50} value was defined as the lowest concentration with 50% radical inhibition or relative reducing power, calculated by linear regression analysis, and expressed as the mean ± SD (n = 3). The linear regression was analyzed by Origin 6.0 professional software. The IC_{50} values with different superscripts (a and b) between acacetin and isoginkgetin are significantly different ($p < 0.05$). Trolox was the positive control.
