# Supplementary material for: 3′,8″-Dimerization Enhances the Antioxidant Capacity of Flavonoids: Evidence from Acacetin and Isoginkgetin
Source: Molecules. 2019 May 28;24(11):2039. doi: 10.3390/molecules24112039 (PMC6600363; doi:10.3390/molecules24112039)
Supplement: Supplementary file 1 [file molecules-24-02039-s001.zip › supplementary-proof/supplementary4.pdf]

Suppl. 4 UV-vis spectra of ABTS$^{+\bullet}$

3’,8”-Dimerization Enhances the Antioxidant Capacity of Flavonoids: Evidence from Acacetin and Isoginkgetin

Xican Li [1,2,*], Xiaojian Ouyang [1,2], Rongxin Cai [1,2], and Dongfeng Chen [3,4,*]

1 School of Chinese Herbal Medicine, Guangzhou University of Chinese Medicine, Waihuan East Road No. 232, Guangzhou Higher Education Mega Center, Guangzhou 510006, China; oyxiaojian55@163.com (X.O.); choi_roy@foxmail.com (R.C.)

2 Innovative Research & Development Laboratory of TCM, Guangzhou University of Chinese Medicine, Waihuan East Road No. 232, Guangzhou Higher Education Mega Center, Guangzhou 510006, China;

3 School of Basic Medical Science & Research Center of Basic Integrative Medicine, Guangzhou University of Chinese Medicine, Guangzhou 510006, China; chen888@gzucm.edu.cn (D.C.)

4 The Research Center of Basic Integrative Medicine, Guangzhou University of Chinese Medicine, Guangzhou 510006, China.

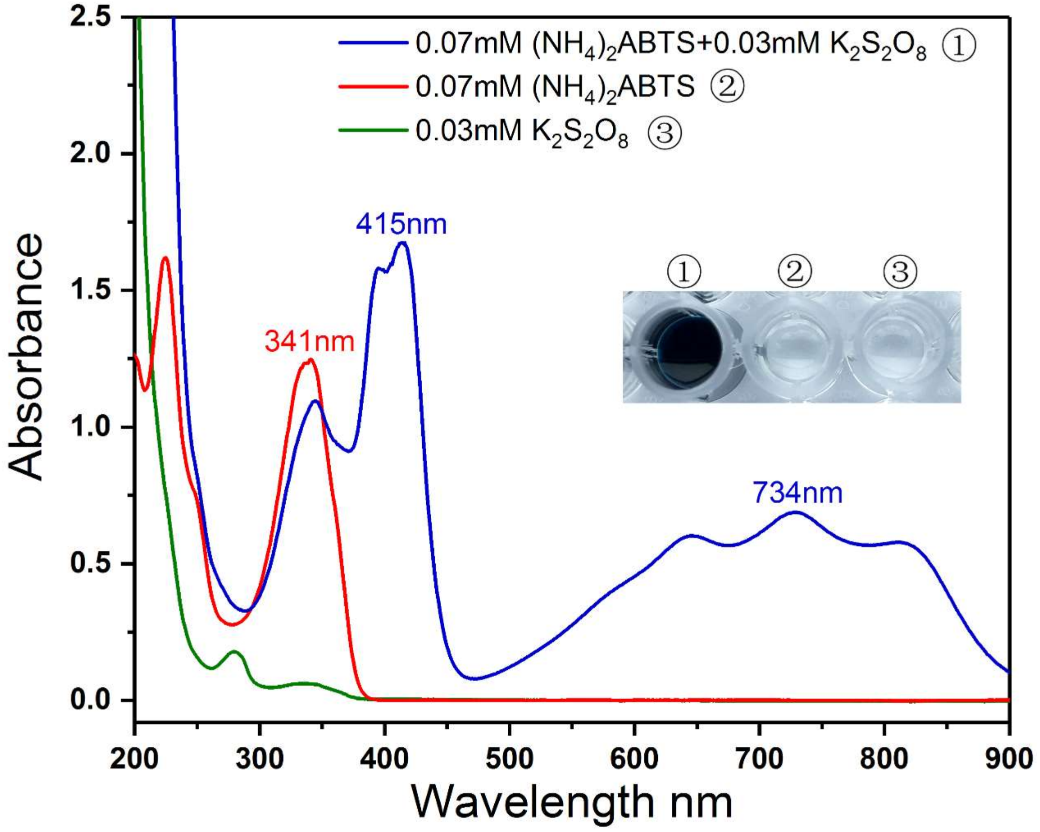

Fig. s4.1 UV-vis spectra of ABTS$^{+\bullet}$
