# Supplementary figures and images for: 3′,8″-Dimerization Enhances the Antioxidant Capacity of Flavonoids: Evidence from Acacetin and Isoginkgetin
Source: Molecules. 2019 May 28;24(11):2039. doi: 10.3390/molecules24112039 (PMC6600363; doi:10.3390/molecules24112039)

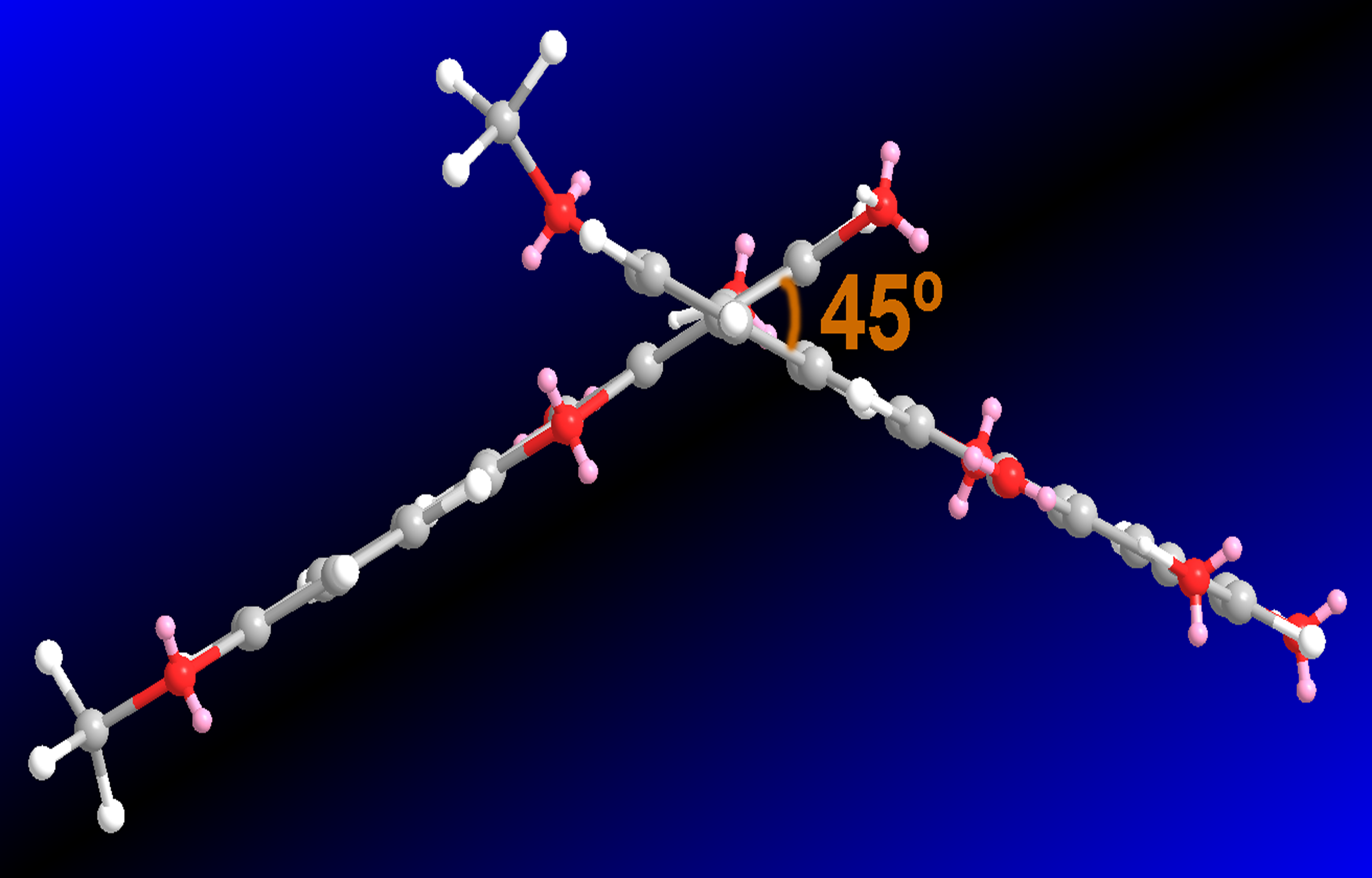

Supplement: Supplementary file 1 [file molecules-24-02039-s001.zip › supplementary-proof/Suppl. 2 Figure S2 dihedral angle 45o+.tif]
